# Epidemiological and clinical characteristics of severe fever with thrombocytopenia syndrome bunyavirus human-to-human transmission

**DOI:** 10.1371/journal.pntd.0009037

**Published:** 2021-04-30

**Authors:** Xinyu Fang, Jianli Hu, Zhihang Peng, Qigang Dai, Wendong Liu, Shuyi Liang, Zhifeng Li, Nan Zhang, Changjun Bao

**Affiliations:** 1 Jiangsu Provincial Center for Disease Control and Prevention (Jiangsu institution of Public health), Nanjing, China; 2 School of Public Health, Nanjing Medical University, Nanjing, China; 3 NHC Key laboratory of Enteric Pathogenic Microbiology, Nanjing, China; University of Texas Medical Branch, UNITED STATES

## Abstract

**Background:**

Severe fever with thrombocytopenia syndrome (SFTS) was listed as one of the most severe infectious disease by world health organization in 2017. It can mostly be transmitted by tick bite, while human-to-human transmission has occurred on multiple occasions. This study aimed to explore the epidemiological and clinical characteristics and make risk analysis of SFTS human-to-human transmission.

**Methods:**

Descriptive and spatial methods were employed to illustrate the epidemiological and clinical characteristics of SFTS human-to-human transmission. The risk of SFTS human-to-human transmission was accessed through secondary attack rate (SAR) and basic reproductive number (R_0_). Logistic regression analysis was used to identify the associated risk factors.

**Results:**

A total of 27 clusters of SFTS human-to-human transmission were reported in China and South Korea during 1996–2019. It mainly occurred among elder people in May, June and October in central and eastern China. The secondary cases developed milder clinical manifestation and better outcome than the index cases. The incubation period was 10.0 days (IQR:8.0–12.0), SAR was 1.72%-55.00%, and the average R_0_ to be 0.13 (95%CI:0.11–0.16). Being blood relatives of the index case, direct blood/bloody secretion contact and bloody droplet contact had more risk of infection (OR = 6.35(95%CI:3.26–12.37), 38.01 (95%CI,19.73–73.23), 2.27 (95%CI,1.01–5.19)).

**Conclusions:**

SFTS human-to-human transmission in China and South Korea during 1996–2019 had obvious spatio-temporal distinction. Ongoing assessment of this transmission risk is crucial for public health authorities though it continues to be low now.

## Author summary

This study is first to explore the epidemiological and clinical characteristics of SFTS human-to-human transmission. We found SFTS human-to-human transmission had obvious characteristic temporal and spatial distinction, which mainly occurred among elder people in May, June and October in the central and eastern China. The secondary cases developed milder clinical manifestation and better outcome than the index cases. The incubation period was 10.0 days (IQR:8.0–12.0). The risk of SFTS human-to-human transmission continues to be low, with the secondary attack rate (SAR) among humans was 1.72%-55.00% and basic reproductive number (R_0_) to be 0.13 (95%CI:0.11–0.16). Being blood relatives of the index case, direct blood/bloody secretion contact and bloody droplet contact had more risk of acquiring infection.

## Introduction

Severe fever with thrombocytopenia syndrome (SFTS) is an emerging infectious disease, which was listed as one of the most severe infectious disease by World Health Organization in 2017 [1]. It was first detected among humans in Henan Province in December 2009 [2]. As of October 2019, 7419 SFTS cases were identified in China, with 355 deaths, who mainly occurred in central and eastern China [3]. SFTS has also reported in South Korea, Japan and Vietnam [4–6]. It characterized by sudden onset of fever, respiratory and gastrointestinal symptoms, followed by the progressive thrombocytopenia and leukocytopenia. A dynamic study was conducted on SFTS cases in Shandong Province reveals 81.35% (48/59) SFTS cases recovered within 2 weeks, and others died due to multiple organ failure (MOF) [7].

SFTS is caused by severe fever with thrombocytopenia syndrome bunyavirus (SFTSV), which can mostly be transmitted by ticks bite (*Haemaphysalis longicornis* and *Rhipicephalus microplus*) [8]. Meanwhile, the secondary cases can be infected through human-to-human transmission of direct contact with blood or bloody secretions bearing SFTSV and probably inhalation of virus-containing droplet and aerosol [9–11]. To date, SFTS human-to-human transmission has been separately reported in South Korea and several provincesin China [9–48]. Nonetheless, no study has depicted overall epidemiological and clinical characteristics of this transmission.

SFTS human-to-human transmission has occurred on multiple occasions. Particularly, the SFTS cases of these transmission events represented a small fraction of the total, and sustained SFTS human-to-human transmission has not been documented. Thus far, no quantitative analysis of SFTS human-to-human transmission risk has been reported. Previous studies have showed that, some blood relatives of SFTS cases and medical personnel were infected among all the contacts, but some were not. Previous studies have assessed the risk factors of SFTS human-to-human transmission and drew inconsistent conclusions, which may be related to the different methods, standards of contact and the small sample size. For example, Gai et.al [10] classified the 63 contacts into 3 types of contact (blood, droplet and possible airborne) and showed that blood contact was the most likely risk factors using multivariate logistic regression analysis. Tang et.al [14] classified the 31 contacts into 4 types of contact (blood, respiratory secretion, urine and feces) and showed that contact with the index patient’s blood on mucous membranes or skin wounds and not wearing personal protective equipment while providing care were significantly associated with disease risk using the χ ^2^ test.

This study first summarized the overall epidemiological and clinical characteristics of SFTS human-to-human transmission in the first and the second part of results section, respectively. Meanwhile, this study assessed the risk of SFTS human-to-human transmission through the secondary attack rate (SAR) and the basic reproductive number (R_0_), and identified associated risk factors in the third part.

## Methods

### Case definition

According to the national guideline for prevention and control of SFTS issued by the Chinese Ministry of Health, suspected SFTS case is defined as the person who presents with acute onset of fever (≥38°C) and other symptoms (e.g., gastrointestinal symptoms, bleeding), epidemiological exposure factors (e.g., being exposed to ticks or similar cases about 2 weeks before illness onset) and laboratory data showing thrombocytopenia or leukopenia. Laboratory-confirmed SFTS case is defined as suspected case who meets one or more of the following criteria: (1) detection of SFTSV RNA, (2) seroconversion or a 4-fold increase of anti-SFTSV immunoglobulin G (IgG) titers between acute and convalescent phase sera, and (3) isolation of SFTSV [49].

In this study, the cluster of SFTS human-to-human transmission was defined as one or more laboratory-confirmed SFTS cases among contacts of suspected/ laboratory-confirmed SFTS cases within 15 days. The index case was identified as the first case in the onset of an epidemiological investigation, who acquired SFTSV infection from an exposure to ticks. The secondary case was identified as the person who got infection from an exposure to the suspected/ laboratory-confirmed SFTS cases, among them, second-generation of secondary case was infected by the index case, and third-generation of secondary case was infected by the second-generation of secondary case.

### Data collection

The databases including PubMed, Web of Science, China National Knowledge Infrastructure (CNKI) and Wanfang Data, were systematically searched for publications about the clusters of SFTS human-to-human transmission. The search was restricted to publications published from January 2010 to December 2019, using a combination of search strings consisting of terms with the exclusion of animal studies (S1 Text).

**Fig 1:**
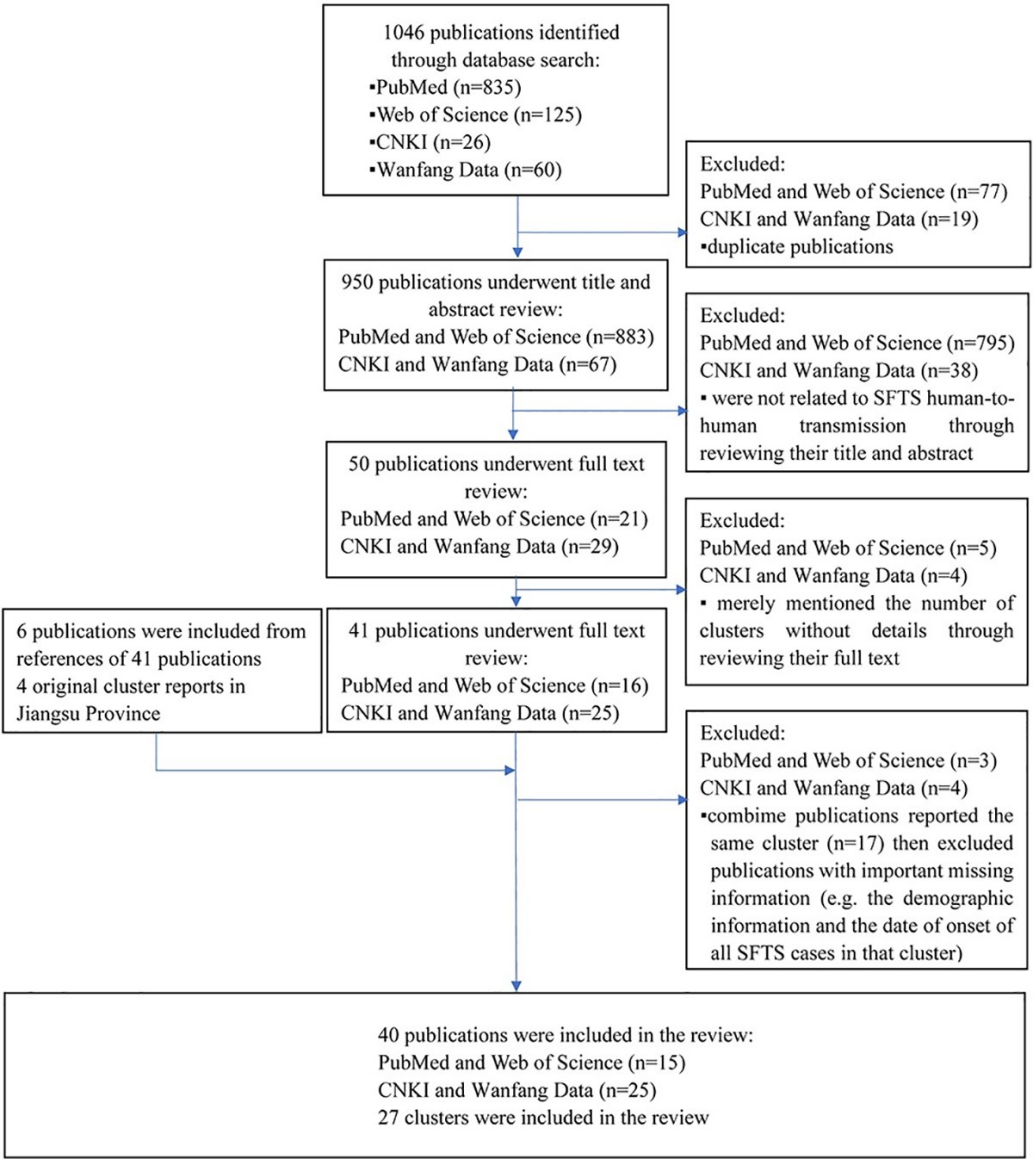
Flow chart of search and selection procedure. Note: n is the number of items in each category.

Publications were selected through four steps, involving duplication, the screening of the title and abstract, the screening of the full text, and further screening during the data extraction phase. The first step excluded duplicate publications. The second step excluded publications which were not related to SFTS human-to-human transmission through reviewing their title and abstract. The third step excluded publications which merely mentioned the number of clusters without details through reviewing their full text. We also checked the references of these publications for relevant publications possibly missed in the search. Additionally, we added 4 original cluster reports in Jiangsu Province. The fourth step combined the results of the publications which reported the same cluster, then excluded publications with important missing information(e.g. the demographic information and the date of onset of all SFTS cases in that cluster) (Fig 1).

The search yielded 1046 publications from 4 databases. A total of 40 publications considered eligible, of which 15 were English publications and 25 were Chinese publications. Finally, 27 clusters of SFTS human-to-human transmission were included in this study (Fig 1).

### Data analysis

Descriptive epidemiological methods and Geographic Information System were employed to illustrate the epidemiological and clinical characteristics of SFTS human-to-human transmission. To determine the difference between the index and secondary cases, Pearson χ^2^ test, Independent Sample T test and Wilcoxon W test were used to compare qualitative variables, normal and abnormal distribution continuous variables, respectively. They test a null hypothesis stating that two sets of identical variables were from the same population.

Subsequently, the risk of SFTS human-to-human transmission was assessed through SAR (i.e. the proportion of individual contacts in whom SFTS developed) and average R_0_ (i.e. the number of people that a SFTS case would infect in a completely susceptible population). The R_0_ of each cluster was first obtained [50], then Kolmogorov-Smirnov test was used to estimate R_0_ distribution, as its overall distribution was unknown, and the average R_0_ and confidence interval were obtained after adjustment for exposed population. If R_0_ <1, transmission chains are not self-sustaining and are unable to generate an epidemic. By contrast, an epidemic is likely to occur whenever R_0_ >1. The mathematical formula was specified as follows:

$$SAR(\%)=\frac{Number\;of\;secondary\;case(s)}{Number\;of\;all\;contact(s)}\times 100$$

$$R_{0}=\frac{Number\;of\;case(s)\;who\;was\;infected}{Number\;of\;case(s)\;who\;infected\;others}$$

Finally, the univariate logistic regression was used to identify the risk factors of SFTS human-to-human transmission, which further incorporated into the multivariate logistic regression for quantitative analysis of adjustment. All-subsets regression was used to fit the optimum model with maximum adjusted R^2^ value. The risks were expressed as odds ratios (OR) and their 95% confidence intervals (CI) were calculated. Wald test was used for test assumptions of parameters, whose assumption was the value of parameter equal to 0. We included the relationship with the index case (medical personnel and blood relatives) and different types of contact as the possible risk factors. All contacts without complete personal protection equipment were classified into 4 types of contact (direct blood/bloody secretion contact, bloody droplet contact, airborne contact and urine/feces/sweat contact). Bloody droplet contact means involving in intubation and/or caring for the index case during bleeding period with close proximity. Airborne contact means staying in a confined space where aerosol may exist for a period of time, i.e., staying in the ward during bleeding period of the index case and (or) funeral room with bleeding corpse.

The methods above were performed in R (version 3.5.1) (S2 Text). The average R_0_ and confidence interval was calculated in Stata (version 12.0). Statistical significance was defined as *P*< 0.05. ArcGIS (version 10.4.1) was used for spatial visualization.

## Results

### Epidemiological characteristics

#### Demographic characteristics

A total of 27 clusters of SFTS human-to-human transmission were reported in China and South Korea during 1996–2019, including 2 clusters of third-generation transmission in China. It contained 138 cases, with an average of 5 cases per cluster (range with 2–12 cases). The index cases were older than the secondary cases with the mean age of 65.4 years (*t* = 4.90, *P*<0.01), however, there were no significant difference in sex ratio between them (*χ*^*2*^ = 1.08, *P* = 0.30). Farmers accounted for 95.83% (23/24) of the index cases and 69.89% (65/93) of the secondary cases (Table 1). There overall 10 asymptomatic infections, of which 7 were second-generation secondary cases, and 3 were third-generation secondary cases. Asymptomatic infections accounted for 7.61% (7/92) and 33.33% (3/9) of all the second-generation and third-generation secondary cases, respectively (*χ*^*2*^ *=* 2.30, *P* = 0.13).

**Table 1:** Demographic and clinical characteristics of cases of SFTS human-to-human transmission in China and South Korea, 1996–2019.

Characteristics	Index cases (n = 27)	Secondary cases (n = 111)	*χ*^*2*^	*t*	*P*
**Demographic features**					
Sex, male	16(n = 27)	65(n = 93)	1.08	N/A	0.30
Age, y	65.35±8.14(n = 26)	51.76±13.35(n = 82)	N/A	4.90	<0.01
Age>65y	12(n = 26)	13(n = 82)	10.18	N/A	<0.01
Farmer	23(n = 24)	54(n = 94)	12.43	N/A	<0.01
Time from onset to treatment, d	4.00±2.81(n = 26)	3.31±1.92(n = 45)	N/A	976.50*	0.62
Time from onset to admission, d	5.57±3.02(n = 26)	3.31±1.92(n = 45)	N/A	3.87	<0.01
**General symptoms**					
Fever	27(n = 27)	96(n = 106)	1.57	N/A	0.21
Fatigue	13(n = 18)	43(n = 64)	0.16	N/A	0.69
Headache	10(n = 17)	37(n = 79)	0.81	N/A	0.37
Myalgia	13(n = 18)	61(n = 85)	0.002	N/A	0.97
**Gastrointestinal symptoms**	23(n = 24)	53(n = 93)	12.65	N/A	<0.01
**Respiratory symptoms**	17(n = 19)	25(n = 62)	14.07	N/A	<0.01
**CNS manifestation**	18(n = 21)	10(n = 70)	38.69	N/A	<0.01
**Hemorrhagic symptoms**	23(n = 24)	17(n = 90)	49.25	N/A	<0.01
**MODS**	16(n = 19)	6(n = 69)	45.31	N/A	<0.01
**Fatal outcome**	27(n = 27)	10(n = 110)	90.89	N/A	<0.01

Note: CNS is central nervous system, MODS is multi organ dysfunction syndrome, and n is the total number of cases for which information is available

* is T value (Wilcoxon W Test), y is year, d is day.

Among 27 index cases, 11 of them had definite history of tick bite. The average time from onset to admission of the index cases was longer than that in the secondary cases (5.6 days versus 3.3 days; *P*<0.01), while there was no difference of the average time from onset to treatment between them (*T* = 976.50, *P* = 0.62). Except for 4 retrospectively confirmed clusters, Laboratory-confirmed SFTS cases and those were confirmed before death accounted for 69.57% (16/23) and 43.48% (10/23) of all the index cases, respectively. Particularly, 3 index cases were misdiagnosed (be diagnosed and be in treatment as other disease rather than SFTS) with influenza and human granulocytic anaplasmosis, enteritis and infectious diarrhea, and blood disorder, respectively (Table 2).

**Table 2:** Line lists of the index cases of SFTSV human-to-human transmission in China and South Korea, 1996–2019.

No.	Reference No.	Location	Date of onset	Date of treatment	Date of admission	Date of death time	Date of confirmed	Time from onset to treatment, d	Time from onset to admission, d	Time from onset to death, d	Time from onset to SFTS diagnosis, d	Other diagnoses
1	[20]	Jiangsu	1996/10/02	1996/10/12	1996/10/14	1996/10/14	N/A	11	13	13	N/A	N/A
2	[11]	Anhui	2006/09/28	2006/09/29	2006/09/29	2006/10/04	N/A	2	2	7	N/A	N/A
3	[11]	Anhui	2006/10/31	2006/11/03	2006/11/03	2006/11/05	N/A	4	4	6	N/A	N/A
4	[9,24]	Jiangsu	2007/04/18	2007/04/19	2007/04/20	2007/04/27	N/A	2	3	10	N/A	N/A
5	[14,26]	Henan	2010/05/20	2010/05/20	2010/05/25	2010/05/30	N/A	1	6	11	N/A	influenza; human granulocytic anaplasmosis
6	[10]	Shandong	2010/09/25	2010/09/28	2010/09/28	2010/10/05	2010/10/05	4	4	11	11	N/A
7	[12,42]	Jiangsu	2010/10/06	2010/10/07	2010/10/13	2010/10/21	2010/10/15	2	8	16	12	N/A
8	[27–29,31]	Shandong	*	2011/10/11	2011/10/11	2010/10/14	N/A	*	*	*	N/A	N/A
9	[13,34]	Hubei	2012/05/06	2012/05/07	2012/05/07	2012/05/12	N/A	2	2	7	N/A	N/A
10	[15,30,43]	Liaoning	2012/06/04	2012/06/06	2012/06/06	2012/06/12	2012/06/11	3	3	9	8	N/A
11	[35]	Zhejiang	2013/05/29	2013/05/30	2013/06/02	2013/06/04	2013/06/04	2	5	7	7	enteritis; infectious diarrhea
12	[16,36]	Shandong	2013/08/25	2013/08/25	2013/08/29	2013/09/04	2013/09/02	1	5	11	9	N/A
13	[32,44]	Anhui	2013/08/27	2013/09/02	2013/09/02	2013/09/07	2013/09/04	7	7	12	9	N/A
14	[17,33,37]	Zhejiang	2014/04/23	2014/04/25	2014/04/29	2014/05/01	2014/05/20	3	7	9	28	N/A
15	[18,38]	Shandong	2014/07/06	2014/07/08	2014/07/13	2014/07/14	2014/07/13	3	8	9	8	N/A
16	[39]	Liaoning	2014/07/28	2014/08/05	2014/08/05	2014/08/07	2014/08/06	9	9	11	10	N/A
17	[40]	Shandong	2015/06/10	2015/06/18	2015/06/18	2015/06/21	2015/06/18	9	9	12	9	N/A
18	[23,41]	Jiangsu	2015/07/10	2015/07/12	2015/07/12	2015/07/18	N/A	3	3	9	N/A	blood disorder
19	[19,46,48]	Jiangsu	2016/05/21	2016/05/21	2016/05/21	2016/05/28	N/A	1	1	8	N/A	N/A
20	JS1	Jiangsu	2016/06/10	2016/06/12	2016/06/16	2016/06/17	N/A	3	7	8	N/A	N/A
21	[45]	Anhui	2016/08/09	2016/08/13	2016/08/14	2016/08/20	2016/08/23	5	6	12	15	N/A
22	[21]	South Korea	2017/09/22	2017/09/24	2017/09/27	2019/10/01	2019/10/01	3	6	10	10	N/A
23	[22]	South Korea	*/10/01	*/10/05	*/10/05	*/10/10	N/A	5	5	10	N/A	N/A
24	[47]	Shandong	2018/05/24	2018/05/30	2018/05/30	2018/06/01	2018/06/01	7	7	9	9	N/A
25	JS2	Jiangsu	2018/06/17	2018/06/17	2018/06/17	2018/07/07	2018/07/06	1	1	21	20	N/A
26	JS3	Jiangsu	2018/06/25	2019/07/02	2019/07/05	2017/07/10	2018/07/11	8	11	16	17	N/A
27	JS4	Jiangsu	2019/08/24	2019/08/26	2019/08/26	2019/08/30	2019/09/14	3	3	7	22	N/A
Mean	N/A		N/A	N/A	N/A	N/A	N/A	4.00	5.57	10.42	12.75	N/A

Note: JS1, JS2, JS3 and JS4 are the 4 original cluster reports in Jiangsu Province

* is missing from publications (We have attempted to contact the corresponding authors for missing information, however, it was missing at the time of the investigation and some of the investigations (e.g. investigation of close contacts) was not carried out at that time), d is day.

#### Temporal and Spatial distribution

**Fig 2:**
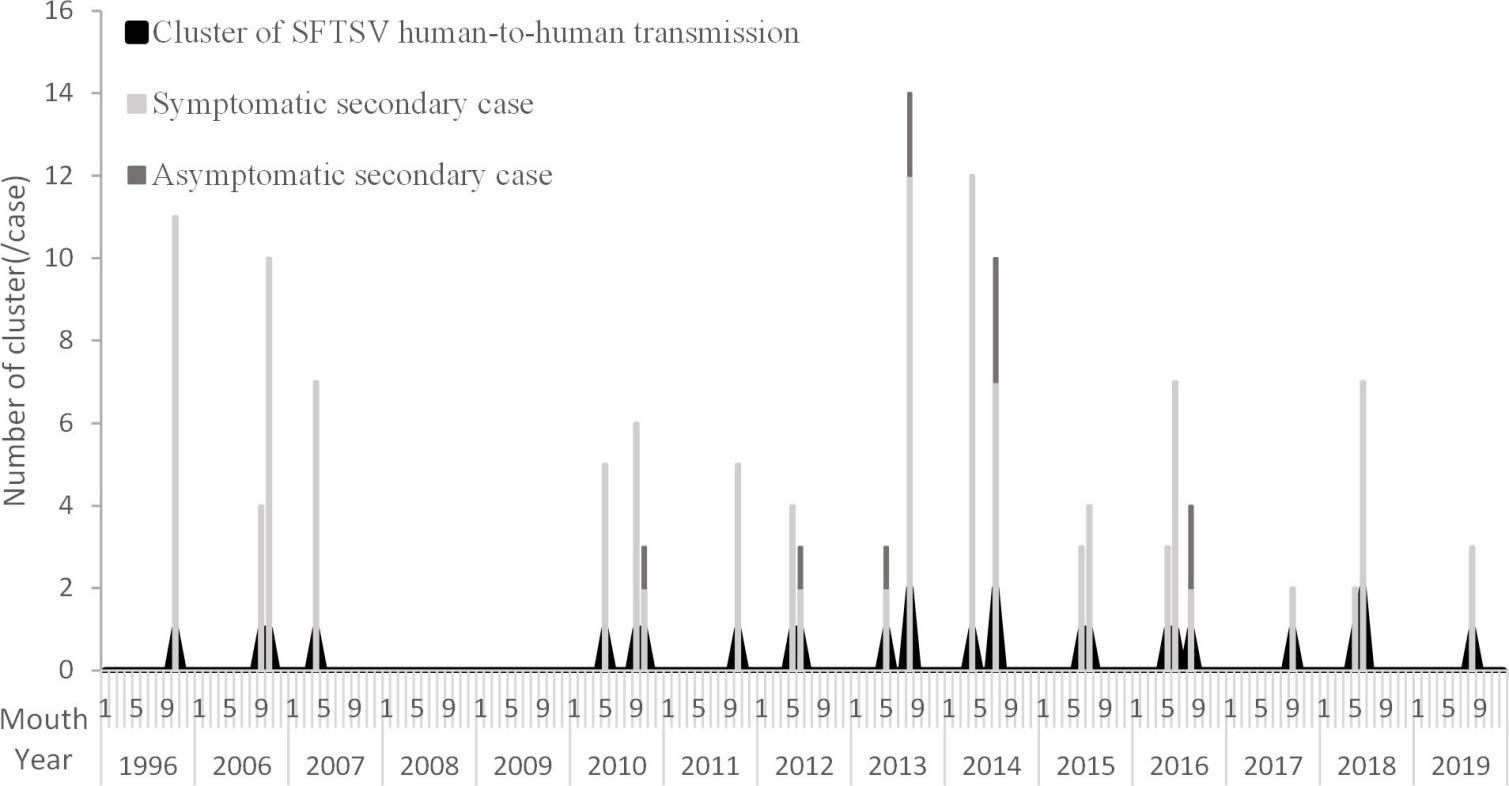
Clusters and cases of SFTS human-to-human transmission in China and South Korea, 1996–2019. Note:The time of 1 nosocomial cluster (6 cases) in South Korea is missing from publications.

Except for 4 retrospectively confirmed clusters (2 in 1996 and 2007 in Jiangsu Province and 2 in 2006 in Anhui Province), 1–3 clusters were reported annually from March to October (mainly in May, June and October) since 2010, when SFTSV was first identified (Fig 2).

A total of 25 clusters occurred in central and eastern China, involving 7 provinces, among which Jiangsu Province reported the most clusters (9 clusters), followed by Shandong Province (6 clusters), Anhui Province (4 clusters), Zhejiang Province and Liaoning Province (2 clusters each), and Henan Province and Hubei Province (1 cluster each). Especially, 2 clusters of third-generation transmission occurred in Shandong Province and Jiangsu Province. Meanwhile, 2 clusters were reported in South Korea.

### Clinical characteristics

There was no statistical difference in general symptoms (fever, fatigue, headache and myalgia) between the index and secondary cases according to Pearson *χ*^*2*^ test. Notably, the index cases had more gastrointestinal symptoms, respiratory symptoms, CNS manifestation, hemorrhagic symptoms and MODS than the secondary cases. All of the index cases eventually died with the average course of illness of 10.4 days, while the mortality of the secondary case was only 9.09% (10/110) (*χ*^*2*^ = 90.89, *P*<0.01) (Table 1).

### Risk analysis

#### Risk assessment

The index cases were highly contagious at least within 2–18 days of onset, leading to secondary infection. The incubation period of the secondary cases was 3–15 days, with the median of 10.0 days (IQR:8.0–12.0). The SAR was ranged 1.72%-55.0%. According to the Kolmogorov-Smirnov test, R_0_ was shown to be Poisson distributed (*Z* = 0.54, *P* = 0.93). The average R_0_ adjusted for exposed population was 0.13 (95%CI:0.11–0.16) (Table 3).

**Table 3:** Risk assessment of SFTS human-to-human transmission in China and South Korea, 1996–2019.

No.	Reference No.	Secondary cases (No.)	Time of exposure since illness onset of index cases min, max (day)	Incubation period min, max, median (day)	Secondary attack rate (Secondary case/Exposure population)	R_0_
1	[20]	10	*, *(n = 0)	*, *, *(n = 0)	23.81% (10/42)	5.0
2	[11]	3	6, 6(n = 3)	7, 9, 7.0(n = 3)	33.33% (3/9)	3.0
3	[11]	9	5, 5(n = 9)	6, 14, 8.0(n = 9)	23.08% (9/39)	9.0
4	[9,24]	6	9, 10(n = 6)	7, 12, 9.0(n = 6)	6.59% (6/91)	6.0
5	[10,14,26]	4	11, 11(n = 4)	8, 10, 9.0(n = 4)	12.90% (4/31)	4.0
6	[10]	5	*, *(n = 0)	7, 15, *(n = 0)	7.94% (5/63)	5.0
7	[12,42]	2	12, 12(n = 1)	10, 10, 10.0(n = 1)	22.2% (2/9)	2.0
8	[27–29,31]	4	5,5(n = 4)	4, 8,7.5(n = 4)	50.00% (4/8)	4.0
9	[13,34]	3	*, *(n = 0)	7, 12, *(n = 0)	4.92% (3/61)	3.0
10	[15,30,43]	2	12, 12(n = 1)	12, 12, 12.0(n = 1)	33.33% (2/6)	2.0
11	[35]	2	2, 2(n = 1)	7, 7, 7.0(n = 1)	25.00% (2/8)	2.0
12	[16,36]	8	12, 12(n = 8)	7, 13, 9.0(n = 8)	20.00% (8/40)	8.0
13	[32,44]	4	7, 8(n = 2)	11, 12, 11.5(n = 2)	13.33% (4/30)	4.0
14	[17,33,37]	11	10, 10(n = 11)	10, 15, 12.0(n = 11)	55.00% (11/20)	11.0
15	[18,38]	5	9, 9(n = 2)	8, 9, 8.5(n = 2)	*	2.0
16	[39]	3	3, 8(n = 30)	8, 12, 11.0(n = 3)	33.33% (3/9)	3.0
17	[40]	2	5, 6(n = 2)	12, 14, 13.0(n = 2)	33.33% (2/6)	2.0
18	[23,41]	3	5, 10(n = 3)	9, 14, 9.0(n = 3)	21.43% (3/14)	3.0
19	[19,46,48]	2	8, 8(n = 2)	8, 12, 10.0(n = 2)	6.25% (2/32)	2.0
20	JS1	6	8, 8(n = 6)	4,14,8.0(n = 6)	31.60% (6/17)	6.0
21	[45]	3	4, 4(n = 1)	10, 10, 10.0(n = 1)	8.82% (3/35)	3.0
22	[21]	1	9,9(n = 1)	12,12,12.0(n = 1)	7.10% (1/14)	1.0
23	[22]	5	5,5(n = 5)	8,11,9.0(n = 5)	20.0% (5/25)	5.0
24	[47]	1	9,9(n = 1)	3,3,3.0(n = 1)	*	1.0
25	JS2	1	18,18(n = 1)	14,14,14.0(n = 1)	1.72% (1/58)	1.0
26	JS3	4	13,16(n = 4)	9,12,10.0(n = 4)	8.89% (4/45)	4.0
27	JS4	2	5,6(n = 2)	10,11,10.5(n = 2)	4.65% (2/43)	2.0
Total	N/A	111	2, 18(n = 80)	3, 15, 10.0(n = 84)	1.72%, 55.00%	N/A

Note: n is the total number of cases for whose information is available

* is missing from publications

#### Logistic analysis

Blood relatives of index case, medical personnel and other persons (including neighbors, body cleaners and those with unclear identities) accounted for 39.64% (44/111), 17.12% (19/111) and 43.24% (48/111) of all the secondary cases. Univariate logistic regression analysis showed that blood relatives had greater risk of infection (OR = 6.35) while the medical personal had lower risk of infection (OR = 0.47). With regard to types of contact, direct blood/bloody secretion contact, bloody droplet contact, airborne contact and urine/feces/sweat contact accounted for 84.76% (89/105), 32.38% (34/105), 66.67% (70/105) and 21.90% (23/105) of the secondary cases. The optimum multivariate logistic regression model (adjusted R^2^ = 0.49) showed direct blood/bloody secretion and bloody droplet contacts were related to the greater risk of infection (OR = 33.81 and 2.27, respectively) (Table 4) (S3 Text).

**Table 4:** Risk factors of SFTS human-to-human transmission in China and South Korea, 1996–2019.

Factors	Contacts (No.)		Attack rate	Univariate analysis		Multivariate analysis	
	Overall	Secondary case		*P*	OR (95%CI)	*P*	OR (95%CI)
Relationship							
Blood relatives of index case	59	44	74.58%	<0.01	6.35(3.26–12.37)	N/A	N/A
	174	55	31.61%				
Medical personnel	85	19	22.35%	<0.01	0.47(0.26–0.83)	N/A	N/A
	225	86	38.22%				
Types of contact							
Direct blood/bloody secretion contact	119	89	74.79%	<0.01	38.01(19.73–73.23)	<0.01	33.81(17.44–65.53)
	221	16	7.24%				
Bloody droplet contact	57	34	59.65%	<0.01	4.41(2.44–7.99)	0.048	2.27(1.01–5.19)
	283	71	25.09%				
Airborne contact	288	70	24.31%	<0.01	0.16(0.08–0.30)	N/A	N/A
	52	35	67.31%				
Urine /Feces/Sweat contact	33	23	69.70%	<0.01	6.31(2.88–13.82)	N/A	N/A
	307	82	26.71%				

## Discussion

To better understand the epidemiological and clinical characteristic of SFTS human-to-human transmission, this study made an analysis of 27 clusters of SFTS human-to-human transmission in China and South Korea during 1996–2019. This study revealed that the clusters of SFTS human-to-human transmission had obvious spatio-temporal distinction. It occurred among elderly people between March and October in the central and eastern China, where SFTS was endemic [3]. The incubation period of SFTS human-to-human transmission was 3–15 days (median of 10.0 days), which is almost consistent with SFTS tick-bite transmission (generally 7–14 days, with average of 9 days) [3].

Farmers are a larger driver in SFTS cases, which may due to farmland and hills being a particularly well-suited habitat for the ticks and farmers had more opportunities to contact with the ticks, though only 11 of them had the definite history of tick bite. Compare to the index cases, the secondary cases developed milder clinical manifestations and better outcomes. It may be explained by the following reasons. Firstly, the diagnosis and treatment of 27 index cases were often delayed, while the secondary cases were relatively timely. In this study, the average time from onset to admission in the index cases was 2.3 days longer than that in the secondary cases. Meanwhile, except for 4 retrospectively confirmed clusters, only 43.48% (10/23) of the index cases were confirmed before death and the misdiagnosis existed, whereas all secondary cases be clearly diagnosed. Early diagnosis and early treatment are important to the clinical outcome of disease, thus the SFTS diagnostic awareness and diagnostic level of medical personnel should be improved. Secondly, the index cases were older than the secondary cases, with poor physical function. Age has been considered significantly related to the disease progression and clinical outcome of SFTS [51].

The risk of SFTS human-to-human transmission continues to be low. Two main reasons may account for this. For one, there was similarities in number (1–3 clusters) and scale (2–12 cases) of the clusters every year, which suggest no change in the risk of SFTS human-to-human transmission. For another, the SAR was 1.72%-55.0% and the average R_0_ of SFTS human-to-human transmission was 0.13 (95%CI:0.11–0.16), which means this transmission was not self-sustaining and is unable to generate a major epidemic. It much less than the R_0_ of Ebola virus disease in multiple outbreaks, though there were differences in model structure and underlying assumptions [52], for example, Chowell et.al estimated R_0_ at 1.33 to 1.35 for the Ebola outbreak in Uganda using SEIR model and employing a Bayesian estimation method. Legrand et.al estimated R_0_ at 2.7 in Uganda using a different modeling approach, which took into account three different transmission settings (community, hospital settings and during funerals).

Blood relatives of the index case had more risk to get infection, who provided more bedside care (39.64%). Meanwhile, genetic susceptibility might be related with SFTSV infection. For example, Sun et al. [53] reported that all three sisters infected with SFTSV and eventually died, while only 4.05% (3/74) of individuals living in the same areas were asymptomatic and others were uninfected. While medical personnel had lower risk to get infection. They get more personal protection equipment even incomplete (face shield and goggles gloves), which could comparatively protect contacts from infection [14,16].

The index cases were highly contagious at least within 2–18 days of onset, leading to secondary infection. Among types of contact, direct blood/bloody secretion contact was the major risk factor for SFTS human-to-human transmission. 84.76% of the secondary cases contact blood/bloody secretion. Particularly, part of secondary cases only contacted with blood of corpse were infected, which indicates that blood remains infectious for a long time even after death of the index cases. Bloody droplet contact might be the risk factor for SFTS human-to-human transmission. Jeong et al. [54] had detected SFTSV from tracheal aspirate of SFTS case. In this study, 89.47% of the index cases had respiratory symptoms and some also had mouth hemorrhagic symptoms, which make SFTSV possible to transmit through bloody droplet with close proximity. And 6 secondary cases who exposed to index patient during endotracheal intubation without direct blood contact got infection in Anhui Province [32]. Urine, feces and sweat contact may not be the risk factor for SFTS human-to-human transmission. None of secondary cases was caused by mere exposure to urine or feces in this study, though SFTSV was detected in urine specimens of SFTS patients in previous study [55]. In addition, 2 secondary cases in Shandong Province only contacted with sweat of the index case and got infection [40]. Thus, exposure to non-hemorrhagic secretions cannot be ruled out as a possible transmission mode and should still be avoided. Airborne cannot be confirmed as the risk factor for SFTS human-to-human transmission in this study, however, 2 secondary cases only stayed in mouring hall but no directly contacted with the index case were infected in Zhejiang Province [17]. SFTS human-to-human transmission also might be linked to the frequency and duration of contact with the index case, individual susceptibility and immune status [9].

This study had a few limitations. Firstly, we excluded the clusters without detail and the SFTS cases who could be either human-to-human or co-exposure to tick. It may underestimate the practical level of SFTS human-to-human transmission. Secondly, the data was mainly collected from previous publications, thus part of information were missing.

In summary, the clusters of SFTS human-to-human transmission in China and South Korea during 1996–2019 had spatio-temporal distinction. Targeted tick removal should be carried out in high-incidence seasons and areas. Meanwhile, the risk of SFTS human-to-human transmission continues to be low, however, ongoing assessment of SFTS human-to-human transmission is crucial for public health authorities.

## Supporting information

S1 Text Search strings used for the systematic literature search. (DOCX)

S2 Text R code. (DOCX)

S3 Text Model equations. (DOCX)
