# Supplementary material for: Epidemiological and clinical characteristics of severe fever with thrombocytopenia syndrome bunyavirus human-to-human transmission
Source: PLoS Negl Trop Dis. 2021 Apr 30;15(4):e0009037. doi: 10.1371/journal.pntd.0009037 (PMC8087050; doi:10.1371/journal.pntd.0009037)
Supplement: S1 Text — (DOCX) [file pntd.0009037.s001.docx]

# Epidemiological and clinical characteristics of severe fever with thrombocytopenia syndrome bunyavirus human-to-human transmission

Xinyu Fang^1#^, Jianli Hu^2#^, Zhihang Peng^1#^, Qigang Dai^2^, Wendong Liu^2^, Shuyi Liang^2^, Zhifeng Li^2^, Nan Zhang^2^, Changjun Bao^1,2*^

1. School of Public Health, Nanjing Medical University, Nanjing, 211166, China.

2. Jiangsu Provincial Center for Disease Control and Prevention (Jiangsu institution of Public health), Nanjing, 210009, China.

# These authors contributed equally to this work.

* Corresponding author: Email: bao2000_cn@163.com, phone number: 025-83759404, Fax: +862583759409.

**Supplement information**

**Search strings used for the systematic literature search**

(this study used the same search strings in four databases)

Search string #1, disease

severe fever with thrombocytopenia syndrome [All Fields] OR new bunyavirus [All Fields] OR SFTS [All Fields] OR SFTSV [All Fields] OR severe fever with thrombocytopenia syndrome virus [All Fields] OR bangyangvirus [All Fields] OR Huaiyangshan banyangvirus [All Fields]

Search string #2, transmission

human-human [All Fields] OR person-person [All Fields] OR cluster [All Fields] OR human-to-human [All Fields] OR person-to-person [All Fields]

Search string #3, limits

1.Human studies

2.Publication date: 2010/01/01-2019/12/31

Number of hits

The combination #1 AND #2 AND #3 of the search strings yielded 1046 hits

Retrieval formula

Search ((((((human-human) OR person-person) OR cluster) OR human-to-human) OR person-to-person)) AND ((((((severe fever with thrombocytopenia syndrome) OR new bunyavirus) OR SFTS) OR SFTSV) severe fever with thrombocytopenia syndrome virus) OR bangyangvirus) OR Huaiyangshan banyangvirus)) Filters: Publication date from 2010/01/01 to 2019/12/31; Humans
