# Supplementary material for: Epidemiological and clinical characteristics of severe fever with thrombocytopenia syndrome bunyavirus human-to-human transmission
Source: PLoS Negl Trop Dis. 2021 Apr 30;15(4):e0009037. doi: 10.1371/journal.pntd.0009037 (PMC8087050; doi:10.1371/journal.pntd.0009037)
Supplement: S2 Text — (DOCX) [file pntd.0009037.s002.docx]

# Epidemiological and clinical characteristics of severe fever with thrombocytopenia syndrome bunyavirus human-to-human transmission

Xinyu Fang^1#^, Jianli Hu^2#^, Zhihang Peng^1#^, Qigang Dai^2^, Wendong Liu^2^, Shuyi Liang^2^, Zhifeng Li^2^, Nan Zhang^2^, Changjun Bao^1,2*^

1. School of Public Health, Nanjing Medical University, Nanjing, 211166, China.

2. Jiangsu Provincial Center for Disease Control and Prevention (Jiangsu institution of Public health), Nanjing, 210009, China.

# These authors contributed equally to this work.

* Corresponding author: Email: bao2000_cn@163.com, phone number: 025-83759404, Fax: +862583759409.

**Supplement information**

**R code**

###t.test###

t.test(age1~case,data=SFTSdata)

t.test(onsetadmission~case,data=SFTSdata)

###wilcox.test###

wilcox.test(onsettreatmnet~case,data=SFTSdata)

###chisq.test###

table1<-(case,sex,data=SFTSdata)

chisq.test(table1)

###logistic analysis###

unimodelA<-glm(case~relatives,

data=SFTSdata,

family = binomial(link =“logit”),

na.action(na.omit))

summary(unimodelA)

unimodelB<-glm(case~medical,

data=SFTSdata,

family = binomial(link =“logit”),

na.action(na.omit))

summary(unimodelB)

unimodel1<-glm(case~blood,

data=SFTSdata,

family = binomial(link =“logit”),

na.action(na.omit))

summary(unimodel1)

unimodel2<-glm(case~droplet,

data=SFTSdata,

family = binomial(link =“logit”),

na.action(na.omit))

summary(unimodel2)

unimode3<-glm(case~Airborne,

data=SFTSdata,

family = binomial(link =“logit”),

na.action(na.omit))

summary(unimodel3)

unimodel4<-glm(case~Urine,

data=SFTSdata,

family = binomial(link =“logit”),

na.action(na.omit))

summary(unimodel4)

multimodel<-glm(case~blood+droplet,

data=SFTSdata,

family = binomial(link =“logit”),

na.action(na.omit))

summary(multimodel)

library(leaps)

leaps<-regsubsets(case~blood+droplet+ Airborne+ Urine,

data=SFTSdata, nbest=4)

plot(leaps,scale="adjr2")
