# Supplementary material for: Epidemiological and clinical characteristics of severe fever with thrombocytopenia syndrome bunyavirus human-to-human transmission
Source: PLoS Negl Trop Dis. 2021 Apr 30;15(4):e0009037. doi: 10.1371/journal.pntd.0009037 (PMC8087050; doi:10.1371/journal.pntd.0009037)
Supplement: S3 Text — (DOCX) [file pntd.0009037.s003.docx]

# Epidemiological and clinical characteristics of severe fever with thrombocytopenia syndrome bunyavirus human-to-human transmission

Xinyu Fang^1#^, Jianli Hu^2#^, Zhihang Peng^1#^, Qigang Dai^2^, Wendong Liu^2^, Shuyi Liang^2^, Zhifeng Li^2^, Nan Zhang^2^, Changjun Bao^1,2*^

1. School of Public Health, Nanjing Medical University, Nanjing, 211166, China.

2. Jiangsu Provincial Center for Disease Control and Prevention (Jiangsu institution of Public health), Nanjing, 210009, China.

# These authors contributed equally to this work.

* Corresponding author: Email: bao2000_cn@163.com, phone number: 025-83759404, Fax: +862583759409.

**Supplement information**

**Model equations**

1.$ln\left( \frac{p}{1-p} \right)=-0.772+1.848x1$

2.$ln\left( \frac{p}{1-p} \right)=-0.480+-0.765x2$

3.$ln\left( \frac{p}{1-p} \right)=-2.550+3.638x3$

4.$ln\left( \frac{p}{1-p} \right)=-1.094+1.485x4$

5.$ln\left( \frac{p}{1-p} \right)=0.722+-1.858x5$

6.$ln\left( \frac{p}{1-p} \right)=-1.009+1.842x6$

7.$ln\left( \frac{p}{1-p} \right)=-2.652+3.521x3+0.822x4$
